# Research matters: How a brother with Down syndrome inspired a PhD in tuberculosis and an ardor for science communication

**DOI:** 10.1371/journal.ppat.1006816

**Published:** 2018-02-08

**Authors:** Meredith Wright

**Affiliations:** Immunology and Microbial Pathogenesis, Weill Cornell Graduate School of Medical Sciences, New York, New York, United States of America; The Fox Chase Cancer Center, UNITED STATES

What do Down syndrome, tuberculosis, and science communication have to do with each other? Not much to the average person, but to me and my scientific journey, they are inextricably linked. I’ve shared my story before in application essays and personal statements, and I share it now publicly in an attempt to emphatically shout that “Yes, research matters!”

My older brother, Billy, has Down syndrome. For those who haven’t had a genetics class in a while, this means that he has three copies of chromosome 21, instead of the usual two. This extra chromosome brings with it developmental and intellectual disability, as well as a propensity for a cheerful disposition, despite a myriad of other health problems. Because of my brother, I’ve known what chromosomes are since I was a small child, and I credit my understanding of the science to my mom and the book *My Sister is Special*, by Larry Jansen. The book tells the story of a boy with a younger sister with Down syndrome and explains in terms that a child can grasp what chromosomes are and how having too many impacts your sibling’s life.

Probably since I was eight years old, I was determined to become a scientist and cure Down syndrome. I worked hard in my science classes growing up, dutifully took AP Biology in high school, and chose Molecular Biology as my major in college. For my junior independent work, I found a lab where I could study reproductive aging and Down syndrome. While completely fascinating, the work proved incredibly difficult—not because of the actual science, but due to the conversations it sparked between my mom and I when I filled her in about my progress. We’d discuss papers I had read that suggested that women who gave birth to children with Down syndrome when they were under the age of 35 (a category my mom fell into) had an increased risk for Alzheimer disease (AD). I’d share lessons I learned in class about how individuals with Down syndrome have an increased risk for seizures and AD too. This led to incredibly emotional conversations in which I struggled to explain the nuances of the science to my mom and learned a lot more about how Billy might change as he aged.

When it came time to begin my senior thesis research, my advisor and I realized that studying Down syndrome exclusively just hit too close to home, and with my well-being in mind, I switched to a project about tau, a protein implicated in AD as well as Down syndrome. At the same time, I was enrolled in a course about the immune system and another about drug discovery, taught by Paul Reider, a chemist who drove home the responsibility that rich countries have to pay attention to diseases plaguing primarily developing countries.

I fell in love with the complexity of the immune system and the millennia-long battle between hosts and pathogens, sparking evolution in both parties in an attempt to survive. I also completely agreed with the notion that affluent countries, as citizens of the world, have a duty to spend time and resources on global health’s biggest challenges. With this in mind, I ticked off the box for “Immunology and Microbial Pathogenesis” on my application to Weill Cornell Graduate School of Medical Sciences.

Fast forward a few years, and I am now a fifth-year PhD candidate studying *Mycobacterium tuberculosis*, the bacterium which causes tuberculosis. I study genes of unknown function, with the hope that our lab might identify potential new drug targets or, at the very least, understand a bit more about the bacterium, which remains one of the world’s deadliest killers. There are many days when experiments haven’t worked out as planned, where it’s easy to feel that the hours spent plating bacteria and getting buffer to a precise pH aren’t helping anyone. In these moments, I try to think of my brother and the various medications he takes. Someone, somewhere, spent years designing these drugs and testing their efficacy and safety. Their hard work gives my brother and countless others seizure-free days and a functioning thyroid. Meanwhile, there remain many unsolved problems in the world of global health. Our basic research matters because of all the people out there suffering from tuberculosis, who are unwittingly spreading it to their caregivers who are too worried to leave their side. Inadequate supplies and education lead to the development of resistant strains of *M*. *tuberculosis*.

And here is where science communication brings my academic journey full circle. All those years ago, reading *My Sister is Special* allowed me to understand what Down syndrome was and the impact it would have on my family. Talking to my mom about my work and coming up with analogies to explain it all trained me in communicating science clearly and accurately. My mom passed away in 2015, and our daily conversations about my lab work are one of the things I miss most about our relationship. They not only helped me practice explaining science to the public, but also allowed her to teach me where various doctors had succeeded and failed in explaining science to my parents over the years. And now, with my work on tuberculosis, I often need to teach my peers that yes, tuberculosis is still a problem globally, even though it is virtually nonexistent in the United States. I wonder how more effective science communication could help curb the development and spread of drug resistant strains of *M*. *tuberculosis*. Communicating research to young and old is a way to enrich lives, nurture understanding about our health and our world, and inspire future generations of scientists. In the final years of my PhD studies, I see that the thread binding together my interest in Down syndrome and tuberculosis all ties in to effective science communication. My hope going forward is to dedicate my career to finding ways to communicate science for the benefit of humanity.

**Image 1:**
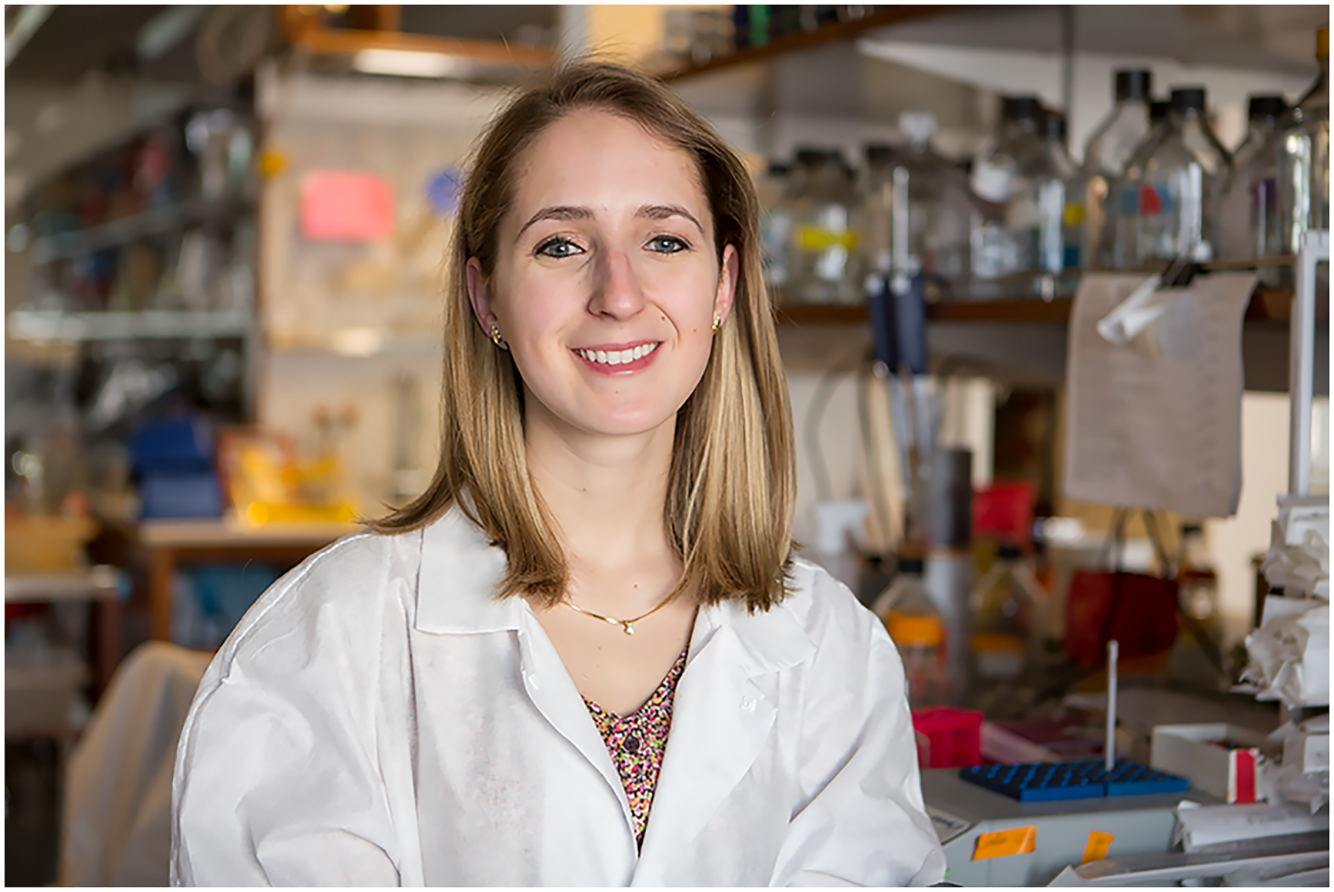
Meredith Wright. Photo by Jon Yu.

